# Supplementary material for: Bi-allelic variants in RNF170 are associated with hereditary spastic paraplegia
Source: Nat Commun. 2019 Oct 21;10:4790. doi: 10.1038/s41467-019-12620-9 (PMC6803694; doi:10.1038/s41467-019-12620-9)
Supplement: Supplementary file 1 — Supplementary Information [file 41467_2019_12620_MOESM1_ESM.docx]

Bi-allelic variants in RNF170 cause hereditary spastic paraplegia

- Supplementary Material –

**Supplementary methods**

**Supplemental information on variant annotation methods**

**Family A -** Genome sequencing of both affected siblings from Familiy A (A.4, A.5) was performed using Truseq PCR-free sample preparation (Illumina), followed by sequencing on a HiSeq X HD v2.5 instrument (Illumina). More than 1,684,998,067 reads with an average read length of 150bp were produced. Data was processed and analysed using the Genesis pipeline^1^. 99.77% of reads could be mapped to the UCSC human reference assembly (hg19); the average read depth was 34.75.

**Family B -** Exome sequencing of one affected sibling (B.4) was performed using the Agilent SureSelect All Exon V6 kit (Agilent) and a HiSeq 2500 (Illumina) platform. Reads were aligned to the human reference genome (UCSC hg19), with Burrows-Wheeler Aligner (BWA,V.0.7.8-r455)^2^. High quality indel and single nucleotide variant calling and annotation were performed using GATK v3.1 using standard filtering criteria (read depth >=10%, genotype quality score >=50).2 Candidate genes were prioritized by searching for homozyogus variants with a minor allele frequency < 0.1% in 1000 in-house ethnically-matched Iranian control exomes, dbSNP, 1000 Genomes and ExAC.

**Family C -** Trio-Exome sequencing of the index case and his parents (C.1, C.2, C.4) was performed using a Sure Select Human All Exon 60Mb V6 Kit (Agilent) for enrichment and the HiSeq4000 (Illumina) platform for sequencing. An average of 135,888,843 reads were produced per sample and aligned to the UCSC human reference assembly (hg19) with BWA v.0.5.8.1 More than 98% of the exome was covered at least 20× and the average coverage was more than 126×. Single-nucleotide variants (SNVs) and small insertions and deletions were detected with SAMtools v.0.1.7. Copy number variations (CNVs) were detected with ExomeDepth^3^ and Pindel^4^. Variant prioritization was performed based on an autosomal recessive (MAF <0.1%) and autosomal dominant (*de novo* variants, MAF <0.01%) inheritance model.

**Family D –** Duo exome sequencing of both affected siblings (D.3, D.4) was done using an xGen Exome Research Panel v1.0 for targeted enrichment and a HighSeq 4000 sequening platform (Illumina). The sequence reads were aligned to the reference genome, hg19, using BWA MEM and underwent duplicate removal (Picard v2.5.0), indel realignment and base quality realignment (GATK) and variant calling (HaplotypeCaller) as recommended in the GATK Best Practices^5^. The detected variants were annotated by ANNOVAR^6^. Variants were filtered to consider only homozygous SNVs and short Indels due to their higher probability of contribution to the disease in this consanguineous family.

**Supplementary Figures**

**
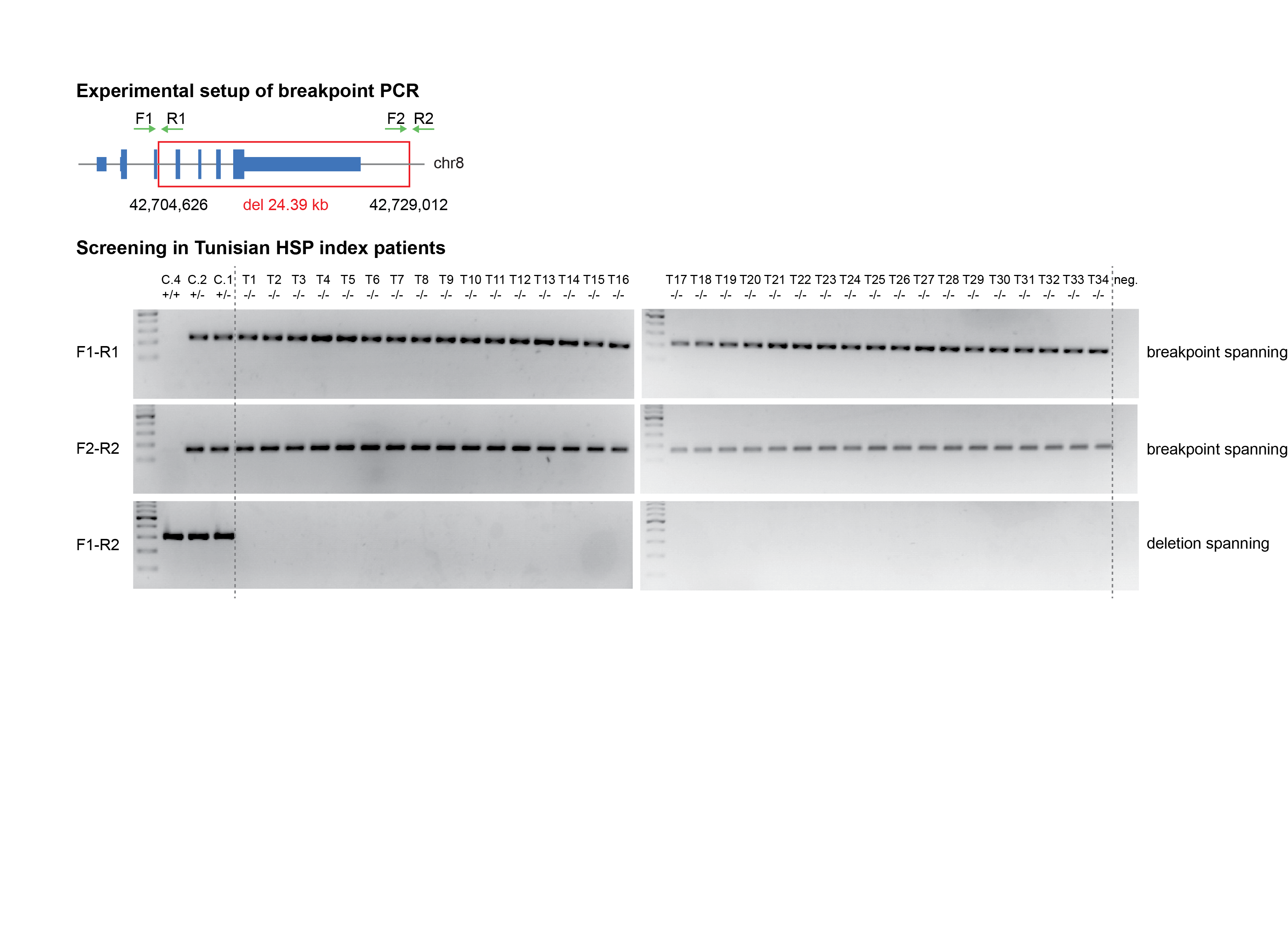
**

**Supplementary Figure 1:** Screening for the chr8:g .42,704,626_42,729,012delinsTTTTGGT mutation in 34 Tunisian HSP index patients of unknown genetic etiology.


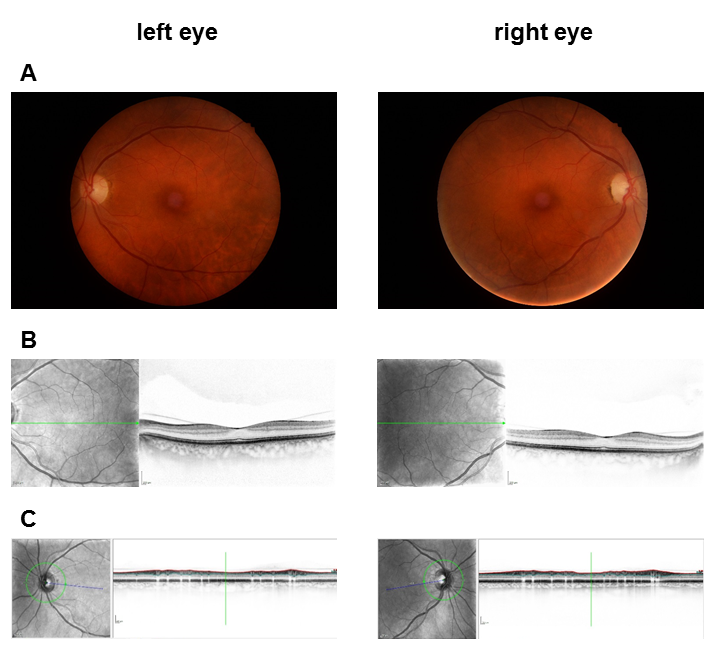
**Supplementary Figure 2: Fundoscopy and optical coherence tomography (OCT) of patient A.4**. Color fundus photography shows mild paleness of the optic nerve head in the temporal sector on both eyes; OCT reveals temporal optic nerve atrophy on both eyes, but otherwise unremarkable retinal structures, in particular no chorioretinal atrophy.


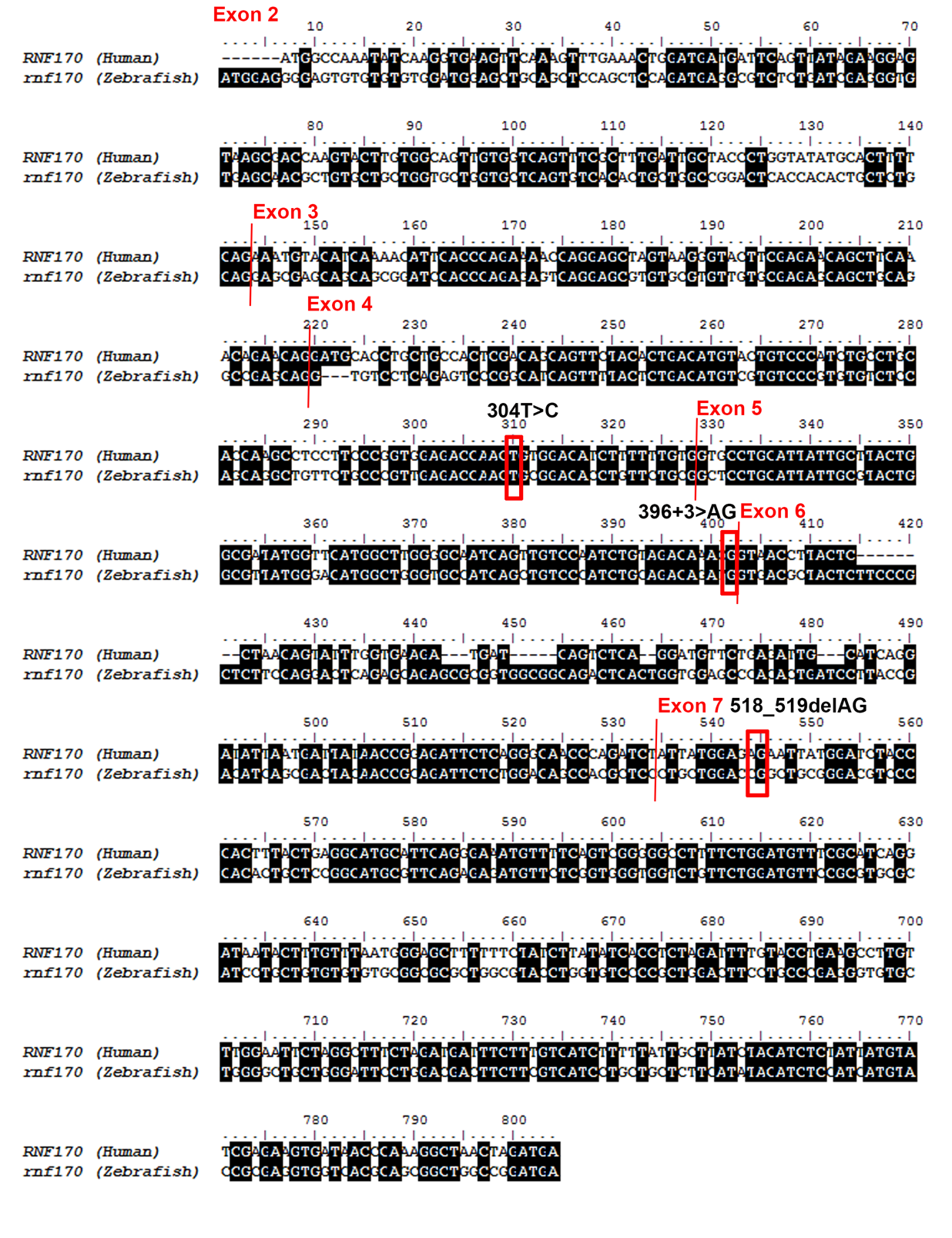


**Supplementary Figure 3:** Alignment of zebrafish *rnf170* coding region with human *RNF170*. Black highlighted nucleotides indicate regions of homology, red bars mark exon boundaries, and red boxes indicate patient mutations.


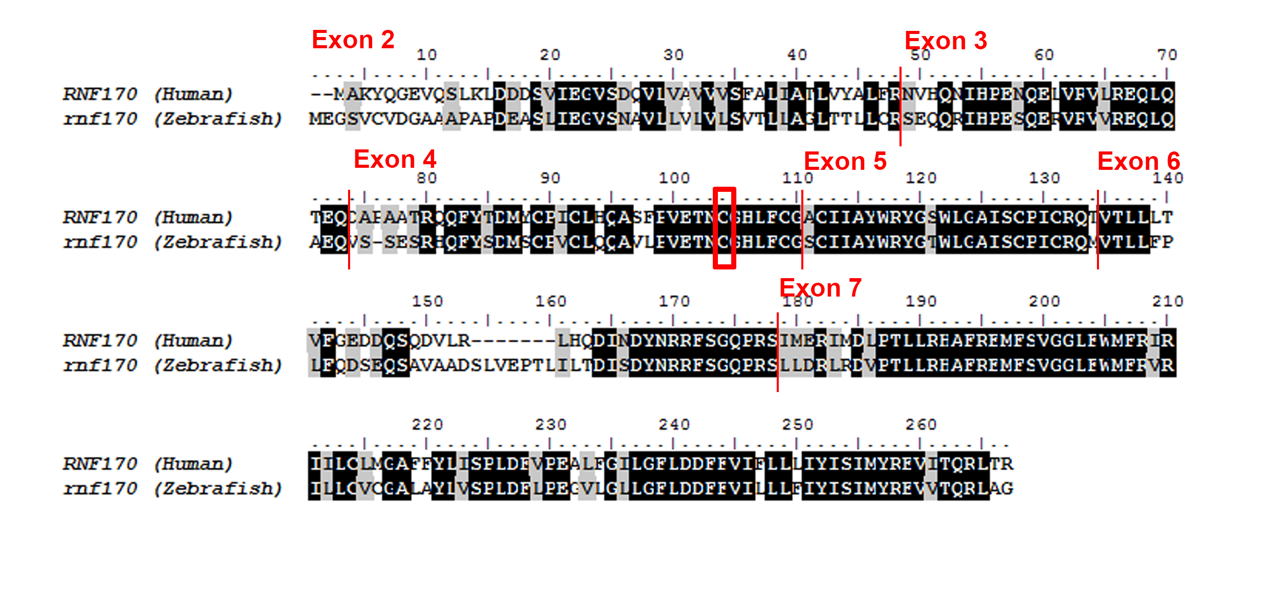


**Supplementary Figure 4:** Alignment of zebrafish Rnf170 protein with human RNF170. Black highlighted amino acids indicate regions of homology, red bars mark exon boundaries, and a red box indicates the patient missense mutation p.Cys102Arg.

**
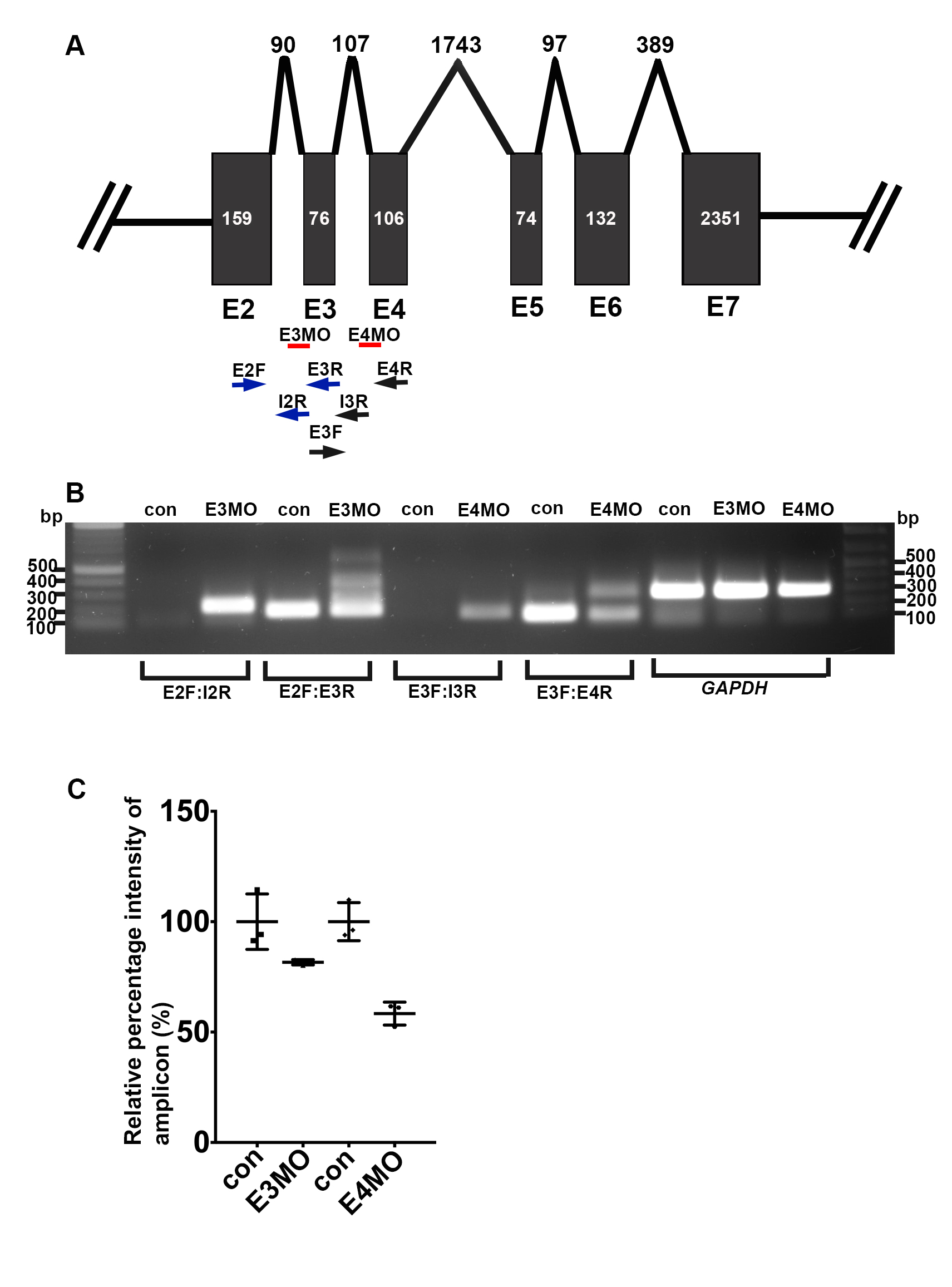
**

**Supplementary Figure 5:** Validation of targeted *rnf170* knockdown by antisense morpholino injections into zebrafish embryos followed by RT-PCR. **(a)** Schematic representation of the two-morpholino target sites (E3MO and E4MO, red bars). Primer sets were designed to validate either E3MO (primers: E2F, I2R, E3R. Blue arrows) or E4MO (primers: E3F, I3R, E4R. Black arrows) by RT-PCR. **(b)** RT-PCR performed on 48 hpf *rnf170* morphant or control morphant embryos (con). Injections of E3MO causes inappropriate splicing of intron 2 and predicted premature stop, as indicated by the presents of an expected 226 bp amplicon in the E3MO lane, compared to its absence in the con lane (primer pair E2F;I2R). Concurrently, amplification using exonic primers (E2F:E3R) results in the expected amplicon of 196 bp in only the con embryos, compared to multiple additional larger products found in the E3MO lane. Similarly, injections of E4MO results in the inclusion of intron 3 and predicted premature stop, as indicated by the presents of an expected 169 bp product (primer pair E3F;I3R) in the rnf170 morphant samples compared to its absence in con embryos. Amplification using exonic primers (E3F:E4R) shows an expected amplicon at approximately 158 bp in control embryos but an additional larger product in the E4MO injected embryos, indicative of the intronic inclusion. Amplification of a 300bp GAPDH amplicon was used as a positive control. **(c)** Quantification of morpholino efficacy by measuring the relative amplicon intensity between expected exonic amplicons (E2F:E3R, E3F:E4R) from control morpholino injected embryos verses morphants. Each band was normalized against GAPDH before relative intensity against control was calculated.


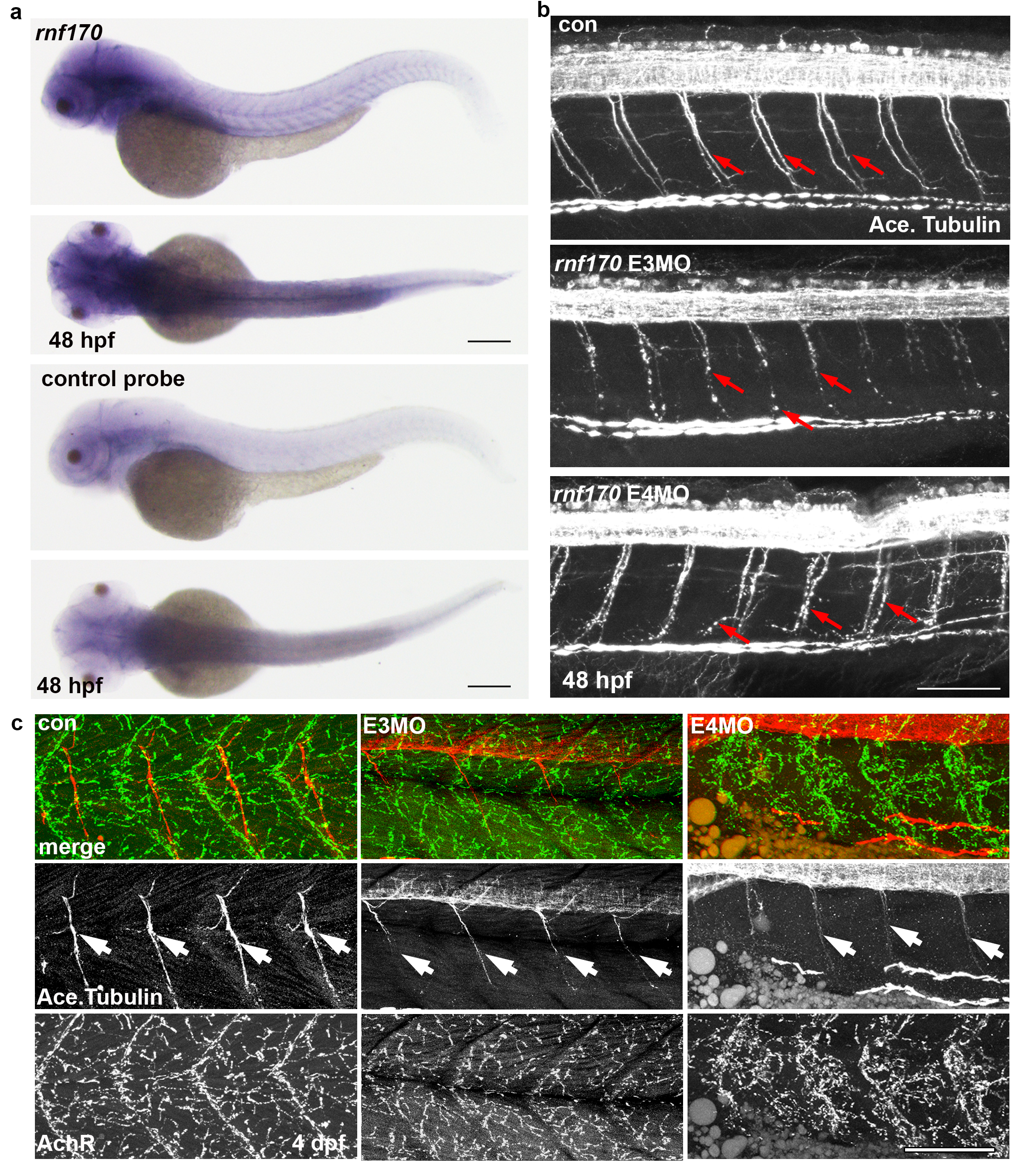


**Supplementary Figure 6: rnf170 is expressed in the developing brain and intersomitic regions and rnf170 knockdown affects motorneuron development.** **(a)** Top two panels show lateral and dorsal views, respectively, of a representative 48 hpf embryo stained using an antisense probe against rnf170 transcript. The two bottom panels show lateral and dorsal views, respectively, of a representative 48 hpf embryo stained using a sense control probe against *rnf170*. Scale bar: 200 µm. **(b)** Staining for acetylated tubulin in larvae 48 hpf shows disorganized caudal primary motorneurons in *rnf170* knockdown embryos. Localisation of antigen signal appeared punctate and intermittent in *rnf170* morphants compared to controls (arrows). Scale bar represents 100 µm. **(c)** At 4dpf *rnf170* morphant verses control MO injected embryos continue to show reduced motorneuron staining in the myotome, whilst acetylcholine receptors (AchR) persists. Motorneurons (arrows) are stained using acetylated Tubulin (red), AchR are marked using bungarotoxin (green). Scale bar represents 100 µm.


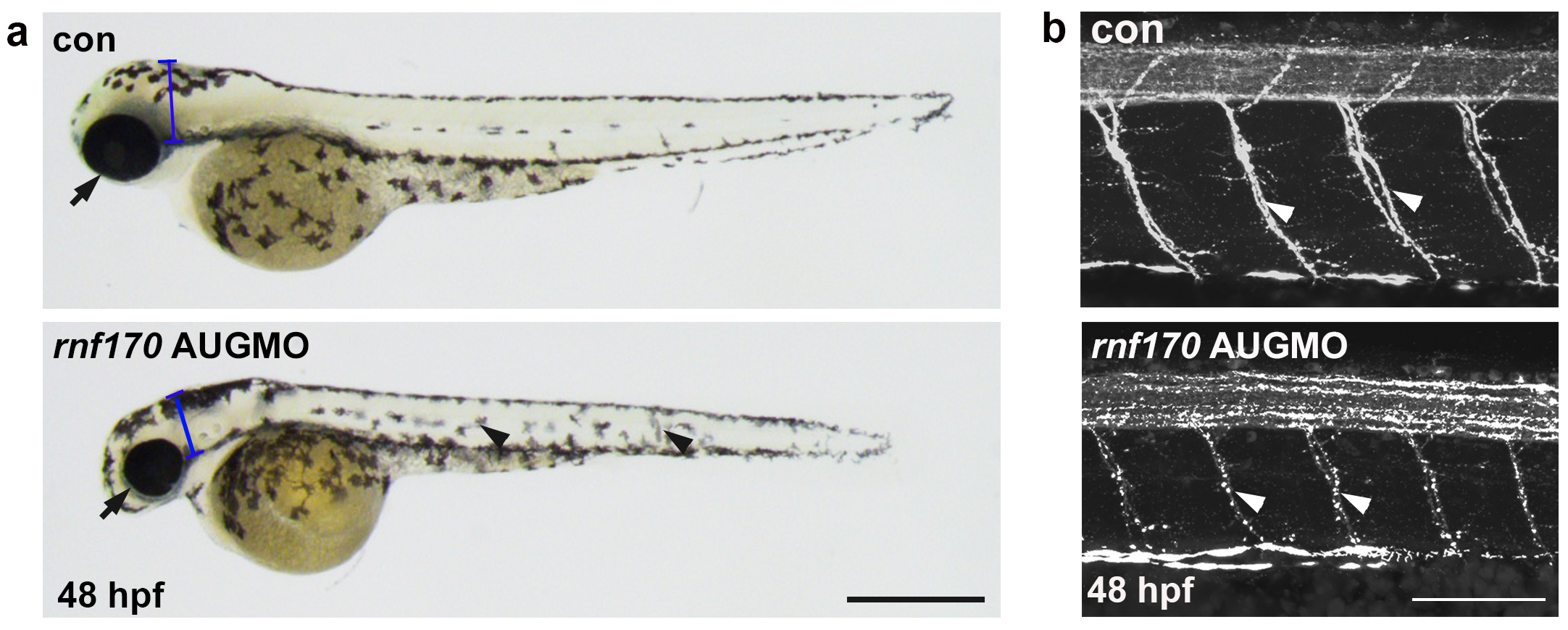


**Supplementary Figure 7: rnf170 knockdown at the translational recognition start site results in a similar phenotype to *rnf170* splice morphants. (a)** *rnf170* AUG morphants display shortened body axis, micropthalmia (arrows), microcephaly (brackets) and alterations in pigmentation (arrow heads). Scale bar represents 500 µm. **(b)** Staining for the axonal marker acetylated tubulin at 48 hpf (arrow heads), *rnf170* AUG morphants display punctate and intermittent antigen localisation when compared to control MO injected embryos. Scale bar represents 100 µm.

**
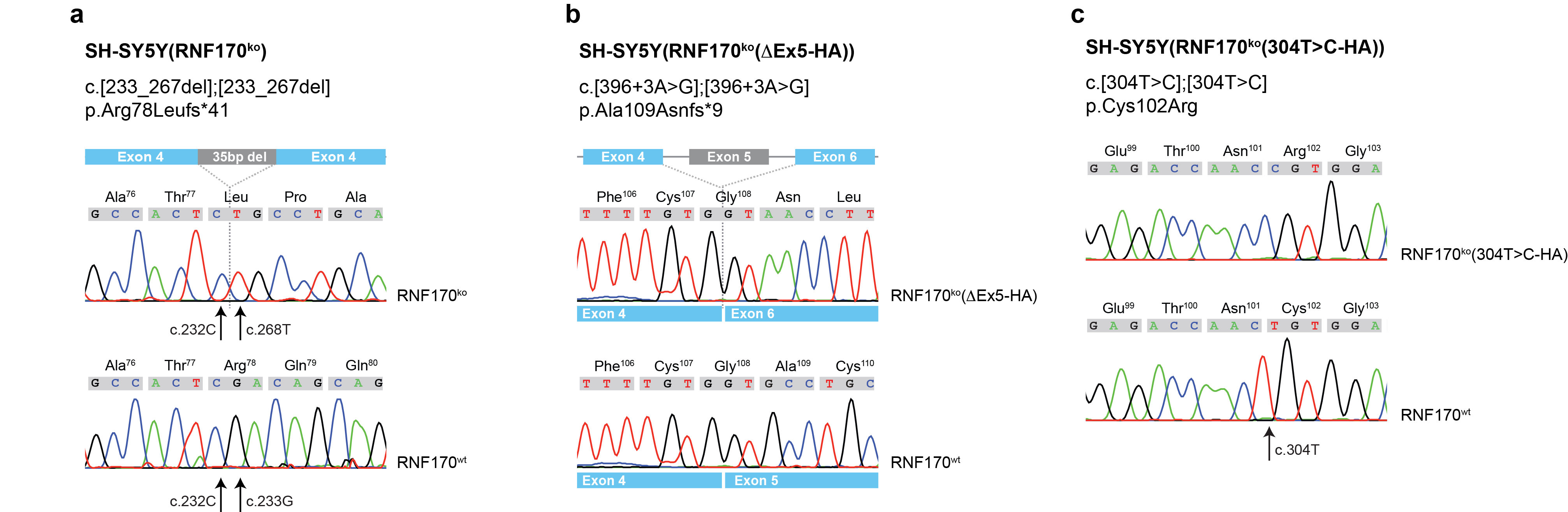
**

**Supplementary Figure 8: (a)** Sanger sequencing confirms presence of a 35bp deletion in the RNF170 gene that was introduced by a CRISPR/Cas9 approach. **(b)-(c)** SH-SY5Y(RNF170^ko^) cells were stably transfected with RNF170 constructs carrying RNF170 mutations.

**Supplementary Tables**

**Supplementary Table 1: Variants in RNF170 identified in the present study**

| **family** | **genomic variant (hg19)** | **zygosity** | **cDNA** | **protein effect** | **mutation type** | **gnomAD alleles** |
| --- | --- | --- | --- | --- | --- | --- |
| *A* | chr8:42720556T>C | hom | NM_030954.3: c.396+3A>G | p.Ala109Asnfs*9 | splice | 1/245854 |
| *B* | chr8:42725165A>G | hom | NM_030954.3: c.304T>C | p.Cys102Arg | missense | 1/246108 |
| *C* | chr8:42704626_42729012delinsTTTTGGT | hom | c.? [delEx4_7] | p.? | CNV | absent |
| *D* | chr8:42711560_42711561delTC | hom | NM_030954.3: c.518_519delAG | p.Arg173Asnfs*49 | deletion | absent |

**Supplementary Table 2: Information on exome sequencing and bioinformatics pipelines**

| Fam-ID | Sequenced individuals | Sequencing center | Instrument | Experiment type | Library/ Exome capture | Reads | Mapped reads | % Mapped | Mean coverage | Coverage 20X | alignment tool | variant caller |
| --- | --- | --- | --- | --- | --- | --- | --- | --- | --- | --- | --- | --- |
| A | A.4, A.5 | Hudson Alpha (Huntsville, Alabama) | Illumina HiSeq X HD v2.5 | WGS | TruSeq DNA PCR-Free Library Prep | 870,404,738 (A.4) / 814,593,329 (A.5) | 868,079,887 (A.4) / 812,703,476 (A.5) | 99.73 (A.4) / 99.77 (A.5) | 34.90 (A.4) / 34.60 (A.5) | 95.5% (A.4) / 93.0% (A.5) | BWA v.0.7.1.2 | Freebayes |
| B | B.4 | Novogene (Beijing, China) | Illumina HiSeq 2500 | WES | Agilent SureSelect Human All ExonV5/V6 | 44,055,430 | 43,991,117 | 99.85 | 99.9% | 94.1% | BWA v.0.7.8-r455 | GATK v3.1 |
| C | C.1, C.2, C.4 | Helmholtz Center Munich, Germany | Illumina HiSeq 4000 | WES (Trio) | Agilent SureSelect Human All Exon V6 | 118,305,358 | 118,076,733 | 99.81 | 126.51 | 98.16% | BWA v.0.5.8 | SAMtools v.0.1.7, ExomeDepth, Pindel |
| D | D.3, D.4 | Yale Center for Genome Analysis | Illumina HiSeq 4000 | WES | IDT xGen® Exome Research Panel v1.0 | 38,541,168(D.1)/41,791,764 (D.2) | 38,514,682(D.1)/41,773,100 (D.2) | 99.93% (D.1)/99.96%(D.2) | 26.789(D.1)/ 29.024(D.2) | 95.8%/97% | BWA  0.7.12-r1039 | GATK v3.6-0-g89b7209  HaplotypeCaller |

**Supplementary Table 3: List of primers used**

| **Primer name** | **Sequence (5’->3’)** |
| --- | --- |
| **Primers for confirmation of gDNA variants (Fig. 1)** | |
| Fam A_F1 | AGGAAGCTACGATCATGCCA |
| Fam A_R1 | AAGGGTTGGCTGGATGAAGT |
| Fam B_F1 | CGTTTACAGTTTGATGAGGGTTACA |
| Fam B_R1 | TTGGTTGACAAGTAGAGCAGGAT |
| Fam C_F1 | GCCAGTCAGTGGTGAGTGAG |
| Fam C_R1 | GTCCATTGGCACCATTTTTC |
| Fam C_F2 | GAAAGAAGCCCATGTTTCCA |
| Fam C_R2 | TTCACCCAGAAAACCAGGAG |
| Fam D_F1 | GCCATGGGTCCTTCTGTTTG |
| Fam D_R1 | CGCGCTAGGTTCTTTGGTTT |
| **Confirmation of splicing defect in Fam A (cDNA) (Fig. 1c)** | |
| Fam A_F_cDNA | CTTCAAACAGAACAGGATGCAC |
| Fam A_R_cDNA | GGGGGCCTTTTCTGGATGTT |
| **qRT-PCR primers (Fig. 1e)** | |
| RNF170_F | GGCAGTTGTGGTCAGTTTCG |
| RNF170_R | CAGGTGCATCCTGTTCTGTTTG |
| RNF10_F | CAC CCA CTG CCA GTC AGG GC |
| RNF10_R | TCC CCG TCG CTG TCC ACA GG |
| RNF111_F | GCAGAATGCAGCAGAAGTTG |
| RNF111_R | CCATTCTTGCAGAAGTGGTTG |
| RPLPO_F | CCCGAGAAGACCTCCTTTTT |
| RPLPO_R | GGGTTGTAGATGCTGCCATT |
| **Confirmation of knockout in SH-SY5Y cells (Fig. 8a)** | |
| SH-SY5Y_F | GTGTTCCAATGTGTGCACCTG |
| SH-SY5Y_R | CCCAAGTATAGCGTTGTTTGCTT |
| **Confirmation of plasmid mutations (Fig. 8b+c)** | |
| plsmd_RNF170_F | GCCACTCGACAGCAGTTCTA |
| plsmd_RNF170_R | GGTCATCTAGTTAGCCTTTGGGTT |

**References**

1. Gonzalez M, Falk MJ, Gai X, Postrel R, Schule R, Zuchner S. Innovative genomic collaboration using the GENESIS (GEM.app) platform. *Hum Mutat* **36**, 950-956 (2015).

2. Li H, Durbin R. Fast and accurate long-read alignment with Burrows-Wheeler transform. *Bioinformatics* **26**, 589-595 (2010).

3. Plagnol V*, et al.* A robust model for read count data in exome sequencing experiments and implications for copy number variant calling. *Bioinformatics* **28**, 2747-2754 (2012).

4. Ye K, Schulz MH, Long Q, Apweiler R, Ning Z. Pindel: a pattern growth approach to detect break points of large deletions and medium sized insertions from paired-end short reads. *Bioinformatics* **25**, 2865-2871 (2009).

5. Van der Auwera GA*, et al.* From FastQ data to high confidence variant calls: the Genome Analysis Toolkit best practices pipeline. *Curr Protoc Bioinformatics* **43**, 11 10 11-33 (2013).

6. Wang K, Li M, Hakonarson H. ANNOVAR: functional annotation of genetic variants from high-throughput sequencing data. *Nucleic Acids Res* **38**, e164 (2010).
