# Supplementary material for: Bi-allelic variants in RNF170 are associated with hereditary spastic paraplegia
Source: Nat Commun. 2019 Oct 21;10:4790. doi: 10.1038/s41467-019-12620-9 (PMC6803694; doi:10.1038/s41467-019-12620-9)
Supplement: Supplementary file 9 — Description of Additional Supplementary Files [file 41467_2019_12620_MOESM9_ESM.pdf]

Title: Supplementary Data 1:

Description: Rare, potentially bi-allelic coding variants identified through whole exome / genome sequencing in families A-D are listed.

Title: Supplementary Movie 1:

Description: Defective motility was observed in rnf170 morphant embryos at 48 hpf, as indicated using a touch response assay. **(a)** Control morpholino injected embryos show the normal response to a gentle prod from a blunt implement, the embryos rapidly swim away.

Title: Supplementary Movie 2:

Description: Embryos injected with E3MO almost completely fail to respond to the stimulus, with the majority of embryos unable to move.

Title: Supplementary Movie 3:

Description: Embryos injected with E4MO also show loss of motility when gently prodded with a blunt implement, embryos fail to swim away.

Title: Supplementary Movie 4:

Description: Control morpholino injected embryos show the normal response to a gentle prod from a blunt implement, the embryos rapidly swim away at 48hpf.

Title: Supplementary Movie 5:

Description: Injections of a translational blocking rnf170 AUG MO results in movement defects when compared to control MO treated embryos (Supplementary Movie 4) at 48hpf.
